# Switch strategies in 2014

**DOI:** 10.1186/1471-2334-14-S2-S4

**Published:** 2014-05-23

**Authors:** Daniel Podzamczer

**Affiliations:** 1 HIV Unit, Infectious Disease Service, Hospital Universitari de Bellvitge, L’Hospitalet, 08907, Barcelona, Spain

## 

Despite great advances in antiretroviral therapy (ART) in the last decade, several limitations remain regarding adverse effects, poor adherence, and drug-drug interactions. Switching ART in stable, virologically suppressed patients with the aim of improving tolerability and convenience is a widely used strategy in clinical practice. Several factors need to be considered when switching from a successful antiretroviral regimen, including previous virologic failure and duration of virologic suppression, genetic barriers to resistance of the new regimen and expected level of adherence. Reductions in the number of pills, drugs, or doses are the most common switching strategies.

Triple therapy regimens including ritonavir-boosted protease inhibitors (PI/r) are the most frequently switched regimens. Thymidine analogues, which are now uncommon in developed countries, have been successfully replaced with non-thymidine analogues in recent years. The main aim of this switch is to prevent or improve abnormal fat redistribution.

Simple one-pill triple regimens such as tenofovir/emtricitabine (TDF/FTC) plus efavirenz (EFV), rilpivirine (RPV) or Elvitegravir/Cobicistat (ELVI/Cobi) may help to improve adherence and reduce some PI/r toxicities. The use of PI/r monotherapy is accepted in EACS Guidelines for selected virologically suppressed patients and may be an option for patients with previous nucleoside analogue toxicities, such as tenofovir-related nephrotoxicity. Raltegravir may be switched to once-daily regimens to improve adherence or it may replace other drugs to avoid interactions in medically complex patients. Unboosted atazanavir is a PI-containing option for patients who do not tolerate ritonavir. Two drug regimens have been assessed in randomized clinical trials and cohort studies with different results because of negative drug interactions leading to unexpected toxicities or low efficacy in some cases.

In summary, many switching options are currently available for virologically suppressed patients. However, although switching strategies may be useful for improving ART, not all regimens used in clinical practice are based on data from randomized clinical trials and not all regimens are suitable for everyone. It is therefore important to individualize treatment, taking into consideration not only patient and drug characteristics but also the best evidence available.

